# Understanding Hurley Stage III Hidradenitis Suppurativa Patients’ Experiences With Pain: A Cross-Sectional Analysis

**DOI:** 10.1177/12034754231188452

**Published:** 2023-07-25

**Authors:** Leah Johnston, Elaine Dupuis, Lauren Lam, Susan Poelman

**Affiliations:** 1 70401 Cumming School of Medicine, University of Calgary, Calgary, Canada; 2 2129 Beacon Dermatology, Calgary, Canada; 3 3158 Department of Dermatology, University of Alberta, Edmonton, Canada

**Keywords:** hidradenitis suppurativa, pain, chronic pain, pain management, quality of life

## Abstract

**Background:**

More than 90% of patients with hidradenitis suppurativa (HS) report that pain interferes with their quality of life (QoL) and pain may have a larger impact on QoL than disease severity alone.

**Objectives:**

The purpose of this study was to understand the impact of pain on the daily lives of patients with Hurley stage III HS.

**Methods:**

This was a single-center, prospective cross-sectional study that was conducted at Beacon Dermatology in Calgary, AB. Patients **≥** 18 years old with Hurley stage III HS in at least one area of the body were prospectively invited to participate in this study. The study consisted of survey questions on patients’ demographic information, past medical histories, HS-related pain histories, and previous therapies for pain management. Additionally, patients completed a series of standardized rating scales on their pain and overall QoL.

**Results:**

Of the 10 patients that participated in the study, 90% (9/10) expressed a desire for more counselling on pain management options. Many patients (8/10, 80%) reported routine use of over-the-counter pain medications and 70% (7/10) used complementary and alternative medicines (CAMs) to manage their pain. Patients’ efficacy ratings of HS treatments in controlling their pain revealed that topical treatments provided minimal or no relief, while surgical interventions had the highest efficacy for reducing pain. Patients’ average worst pain over the preceding 24 hrs was 6.3 +/- 2.5 (2-9) on the Numerical Rating Scale for pain and the mean Dermatology Life Quality Index score was 19.5 +/- 8.2 (5-29).

**Conclusions:**

Patients with Hurley stage III HS report high levels of daily pain and QoL impairment and many individuals use over-the-counter treatments and CAMs to manage their pain. Physicians involved in the care of HS patients should consider implementing routine counselling on pain management into their clinical practices, especially for patients with severe HS.

## Introduction

Hidradenitis suppurativa (HS) is a chronic, inflammatory skin disease that can be extremely painful and debilitating for many patients. HS is characterized by inflammatory nodules and abscesses that develop in intertriginous areas, such as the axilla, inframammary folds, groin and buttocks.^1^ Over time, sinus tracts can form underneath the skin between individual HS lesions, leading to significant pain, drainage, scarring and mobility restriction.^1^ As a result, more than 90% of HS patients report that pain interferes with their quality of life (QoL).^2^ Previous studies have demonstrated that pain may have a more substantial impact on QoL than HS severity alone.^3-5^ Pain can adversely impact sleep, ability to attend work or school, social relationships, and other activities of daily living.^6^

However, pain is a frequently overlooked aspect of HS management and there are many barriers to effective pain management in HS. Often, it may take years for patients to receive a formal diagnosis of HS after symptom onset, due to a lack of awareness of HS within the medical community.^7-9^ In the interim, patients may receive suboptimal management, leading to disease progression.^8^ Once a diagnosis of HS is made, support offered for pain management may also be limited. Factors that limit patients’ access to pain management include lack of provider training and comfort in prescribing pain medication, poor access to outpatient clinics during flare-ups, and patients’ fears of stigmatization and being labelled as ‘drug-seeking’ by healthcare providers (HCPs).^10^ Suboptimal pain management in outpatient settings may lead to frequent emergency department visits and an increased risk of substance use disorder.^11-13^ Thus, it is essential that physicians provide thorough counselling on pain management to HS patients who are significantly impacted by pain.

HS patients may experience both nociceptive and neuropathic pain.^3,13,14^ Nociceptive pain occurs during acute flare-ups of HS, due to tissue damage and inflammatory cytokine release in HS lesions, with subsequent activation of nociceptors in the skin.^13,14^ Nociceptive pain tends to be well-localized to the area of skin injury and presents with ‘throbbing,’ ‘aching’ or ‘constant’ pain at the area of tissue damage. Neuropathic pain tends to be more chronic in nature and is the result of direct nervous system injury, leading to pain that may be described as ‘burning,’ ‘tingling’ or ‘shooting’ and it may also present with allodynia or numbness.^13,14^ This type of pain often occurs in the absence of noxious stimuli and may be diffuse or may be localized to the region that is innervated by a damaged nerve. Patients may respond differently to pain medications based on whether they have nociceptive, neuropathic, or mixed-type pain.^3^ Therefore, it is important to assess both quantitative intensity of pain and qualitative symptoms when taking a pain history from HS patients.

The purpose of this study is to highlight the challenges faced by HS patients with severe disease with respect to pain from their condition and to understand if pain management is an unmet need from the perspectives of a group of Canadian HS patients with Hurley stage III disease.

## Methods

### Study Design

This was a single-center study that utilized a cross-sectional design. Eligible participants were prospectively enrolled and asked to complete a paper-based survey, which included a standardized set of questionnaires. A retrospective review of patients’ electronic medical record charts was also completed. Ethics approval was obtained from the University of Calgary’s Conjoint Health Research Ethics Board.

### Participants

Patients with Hurley stage III HS who had ≥1 active HS lesion in at least one area of the body and were over the age of 18 years were prospectively invited to participate in the study during routine clinic appointments. All participants were patients of a dermatologist at Beacon Dermatology in Calgary, Alberta, Canada. Patients who attended an appointment at the clinic between September and December 2022 were provided with an information handout on the study and asked to follow-up with the study co-ordinator (LJ) if they were interested in study participation. Participants were informed that study participation was voluntary and written informed consent was obtained for all participants. Participants were provided with paper copies of the survey and were instructed to either complete the survey on-site or scan and email the completed survey to the study co-ordinator.

### Survey on Past Medical History, HS Treatments and Pain History

The survey was comprised of 4 main sections: (1) Demographic Information and Medical History (2) Previous HS Treatments (3) HS-Related Pain History and Pain Management (4) Standardized Pain and Quality of Life Rating Scales. The first 3 sections included a combination of open-ended questions and Likert rating scales on past medical history, co-morbidities, HS severity and symptoms, previous HCPs seen for their HS, and previous and current treatments for HS lesions and for managing pain. Patients’ self-reported medical histories and physical exam findings were verified using data from previous clinic visit notes in Beacon Dermatology’s electronic medical record system.

Participants were asked to rate both changes in HS disease activity and changes in pain for a set of common treatments for HS as well as changes in pain levels for a set of pain remedies. Efficacy in improving symptoms was rated on a scale of 1-5, with 1 = no change in symptoms, 2 = minimal improvement, 3 = moderate improvement, 4 = excellent improvement, 5 = complete improvement.

### Standardized Rating Scales

In Part 4 of the survey, participants were asked to complete a series of standardized rating scales to capture the impact of their HS on their overall QoL and provide data on the quantitative severity of their pain and specific pain sensations that they experience.

The Numerical Rating Scale (NRS) for pain and Dermatology Life Quality Index (DLQI) are commonly used in clinical trials of biologic therapies for HS.^14-16^ In the NRS, patients are asked to provide a quantitative measure of their pain during the past 24 hrs on a scale that ranges from 0 to 10, with 0 indicating no pain and 10 indicating the most severe pain possible.^14^ The DLQI is used to assess QoL impairment for a variety of dermatologic conditions.^15^ Questions included in the DLQI do not ask about the impact of specific symptoms on patients’ daily lives and only provide a general overview of the impact of HS on QoL.

The Brief Pain Inventory (short form) is used to evaluate both pain intensity and the impact of pain on daily functioning.^17^ The BPI measures interference of pain with general activity, mood, walking ability, work, housework, social relationships, sleep, and enjoyment of life. The Short-Form McGill Pain Questionnaire 2 (SF-MPQ-2) asks patients to rate the intensity over the preceding week for a series of 22 different descriptive terms for pain sensations.^18,19^ SF-MPQ-2 items are rated on a scale of 0 (none) to 10 (worst possible level). Each descriptive item is associated with one of four pain categories: 1) Continuous 2) Intermittent 3) Affective and 4) Neuropathic.

### Outcome Measures

The primary outcome measures aimed to capture both quantitative and qualitative aspects of HS patients’ pain. Additionally, information was also gathered on the impact of pain on HS patients’ daily lives, the impact of previous HS treatments on their pain, the efficacy of over-the-counter and prescription pain therapies, and previous experiences seeking support for pain management from a HCP.

### Statistical Analysis

Data from standardized surveys and patient charts in Beacon Dermatology’s electronic medical record system (Healthquest) was compiled into a centralized, secure database. Descriptive analysis was used to summarize study data and analyzed data were reported in data tables. Categorical data were reported with frequencies of each score and relative frequencies. Continuous variables were reported with statistics such as mean, median, mode and/or standard deviation.

## Results

A total of 10 patients with Hurley stage III HS met inclusion criteria for participation and were enrolled in the study. Patient characteristics, clinical features of HS, and HS symptoms in these patients is summarized in Supplemental Table 1. All patients were returning patients who had been seen previously by a dermatologist at Beacon Dermatology. Six patients presented for routine follow-up visits, while four patients had booked urgent fit-in appointments for management of HS flare-ups.

The study cohort included 8 females and 2 males, with a median age of 37 years (range: 28-51 years). The mean age of onset of HS was 19.6 years (range: 8-44 years) and at the study enrolment visit, patients had lived with HS for an average total duration of 18.7 years (range: 2-34 years). All patients had HS in 2 or more areas of the body, with a median of 3 different anatomic locations affected by HS lesions. The most frequently affected site was the groin (9/10 patients), followed by the axilla (8/10), inframammary folds and chest (7/10), buttocks (5/10), abdominal pannus (3/10), inner thighs (2/10), and the anterior neck in one patient.

The patient-reported frequency of flare-ups was weekly in five patients, weekly to monthly in two, monthly in two, and every few months in one patient. All patients (10/10, 100%) reported both pain and pruritus as symptoms of their HS.

Thirty percent of patients had previously discussed pain management with a HCP, while 70% had not received any pain management counselling (Supplemental Table 2). However, 90% (9/10) of patients expressed a desire for more counselling on pain management options. Three patients (30%) had been previously prescribed pain medication by a physician. Eighty percent of patients (8/10) reported the use of over-the-counter pain medications and 70% (7/10) indicated that they routinely use complementary and alternative medicines (CAMs) to manage their pain. Specific CAMs included cannabis products (6/10, 60%) and dietary supplements (4/10, 40%).

HS patients’ self-ratings of the efficacy of different treatments for managing both their HS lesions and their pain are depicted in Supplemental Table 3. Topical therapies, including topical antibiotics, resorcinol, and antiseptic washes, had the lowest patient ratings for improvement in pain, with median scores ranging from 1 to 2. Surgical procedures, including deroofing and incision and drainage, had the highest ratings for improvement in pain, with a median rating of 4 (excellent improvement) for both procedures. Most patients (90%, 9/10) were currently on or had previously utilized one or more biologic therapies, which had a median pain improvement rating of 3 (moderate improvement). Oral antiandrogens and intralesional corticosteroids also had moderate median ratings for pain improvement.

One patient reported use of a gabapentinoid, and two patients had been previously prescribed opioids during acute HS flare-ups or following surgical procedures. No patients reported use of a selective-serotonin reuptake inhibitor or a selective-norepinephrine reuptake inhibitor. Patients reported pain relief scores for the following over-the-counter treatments and CAMs: topical lidocaine creams, acetaminophen, non-steroidal anti-inflammatory drugs such as ibuprofen, hot compresses, and cannabis. Patients’ mean and median ratings for improvement in pain were in the minimal to moderate improvement range for these therapies.

HS patients’ scores on standardized pain and QoL rating scales are depicted in Supplemental Table 4. The mean NRS score for worst pain over the past 24 hrs was 6.3 +/- 2.5 (2-9) and patients’ least pain was 3.8 +/- 2.4 (0-7). Patients’ average daily pain was 4.9 +/- 2.4 (0-8) and their current pain while completing the survey was 4.9 +/- 2.9 (0-9). The mean DLQI score was 19.5 +/- 8.2 (5-29) and 90% of patients’ DLQI scores corresponded to the ‘large’ (4/10, 40%) or extremely large (5/10, 50%) impact on QoL categories for DLQI scores. The mean score on the BPI interference items was 5.6 +/- 3.0 (0.6- 10), which corresponded to the ‘high level of interference with daily activities’ category.

The mean total score on the SF-MPQ-2 was 3.4 +/- 2.1 (0.6-6.3). Mean scores on the SF-MPQ-2 subdomains were 3.1 +/- 2.1 (0.3-6.3) for continuous pain, 3.5 +/- 2.2 (0.3-6.0) for intermittent pain, 2.9 +/- 1.9 (0.3-6.0) for neuropathic pain, and 4.4 +/- 4.1 (0.0-10.0) for the affective dimension of pain. The top five rated descriptive terms for pain were ‘tender,’ ‘itching,’ ‘pain caused by light touch,’ ‘stabbing pain,’ and ‘tiring-exhausting’ (Supplemental Table 5).

## Discussion

The results from this study provide insight into HS patients’ experiences living with pain, navigating the healthcare system, self-treating their pain, and seeking medical support for pain management. All patients indicated that pain is a frequent symptom of their HS. Average daily pain levels were in the moderate severity range, indicating that patients are consistently burdened by pain. Additionally, the results supported high levels of impairment in patients’ QoL and their ability to complete activities of daily living. The results from the SF-MPQ-2 did not show a predominance for either nociceptive or neuropathic pain, indicating that patients may experience both types of pain on a regular basis. Interestingly, the two highest rated pain descriptor terms in this study, ‘tender’ and ‘itching,’ were also two of the highest-rated terms in a recently published study that evaluated patients’ ratings of pain on the Pain Quality Assessment Scale.^10^

Only a few patients had previously received a prescription for pain medication. This study revealed a substantial discrepancy between the number of patients who had previously discussed pain management for their HS with a HCP (30%) and the number of patients who desired more counselling on this topic (90%). This indicates that patient counselling on pain management may be an unmet need in the HS patient population, particularly for patients with severe disease. A recent study that interviewed 12 HS patients on their treatment priorities showed a similarly high desire for pain management, with 83% (10/12) of patients mentioning that pain control was an important goal in their treatment plans.^20^ However, only 7/16 (44%) of dermatologists mentioned pain management in their interview answers, highlighting the importance of focused counselling on pain management when discussing treatment plans with HS patients.

Patient ratings of the efficacy of HS treatments in controlling their disease activity and pain revealed that topical treatments provided minimal or no improvement of both outcomes. Biologic therapies, intralesional corticosteroids, and oral antiandrogens provided the most pain relief out of the medical therapies that were included in the survey. However, ratings for these medication classes indicated that many patients experienced only minimal to moderate relief of their pain with medical therapies. This data is consistent with results from clinical trials for biologic therapies, which showed that patients with Hurley stage III HS had poorer responses to biologics due to their advanced disease.^21^ In this study, surgical interventions for HS, including incision and drainage and deroofing, had the highest patient-reported efficacy in reducing pain severity. These results highlight the importance of prioritizing surgical procedures in the management of patients with Hurley stage III HS, as they may have several areas of skin that are covered with sinus tracts, which are often refractory to systemic medical therapy alone.^22^ At Beacon Dermatology, a modified deroofing surgical technique is utilized to thoroughly excise the fibrotic tissue lining sinus tracts, providing a low rate of disease recurrence.^23^ However, wait times for surgical procedures may be months’ long in some geographic areas and the number of areas that can be treated during one surgical appointment may be limited. Therefore, interim pain management may be essential for improving patients’ QoL prior to their scheduled surgeries.

A notable consequence of the lack of provider-initiated pain management counselling and limited efficacy of medical therapies in improving HS patients’ pain is that many patients use over-the-counter medications and CAMs to self-manage their pain.^24,25^ However, this study found that many self-initiated treatments had minimal success in improving patients’ pain. Similarly, a 2022 survey also found that most CAMs used by HS patients only had mild to moderate efficacy.^25^ Additionally, previous studies have found that patients with poorly controlled pain may use dangerously high doses of over-the-counter therapies or may misuse prescription medications that were prescribed for a different indication or to a family member.^10,24^ This further highlights the importance of discussing pain management with HS patients, to increase patients’ access to pain management options that are both safe and effective.

A limitation of this study was the small sample size of the study cohort (n = 10). To understand the challenges faced by patients with the most severe stage of HS, the study was restricted to patients with Hurley stage III disease, which comprises only about 4% of the total HS patient population.^26^ The sample size was also limited by single-center recruitment and prospective enrolment of patients who attended clinic visits during the study enrolment period. Additionally, although previous studies have found that pain was highly correlated with disease severity, inclusion of patients based on clinical Hurley staging alone may be a less accurate representation of the pain experienced by patients with acutely active disease.^27^ Recent studies have found ultrasonography may be beneficial in detecting sonographic features that are associated with increased disease activity and pain, such as intense vascularization, and classification of Hurley stage based on ultrasonography may result in upstaging compared to clinical staging alone.^28-30^ Finally, as this study predominantly focused on HS patients’ experiences with pain, the impact of other symptoms on patients’ QoL, such as pruritus and wound drainage, was not explored in-depth. However, all patients in this study reported experiencing pruritus as a HS symptom and this attribute had the second highest severity rating out of all 22 pain descriptors on the SF-MPQ-2. A previous systematic review of itch in HS found that between 35-82.6% of HS patients experienced pruritus on a regular basis and that pruritus has been associated with impaired QoL and psychiatric co-morbidities in HS patients.^31^ Further study on the impact of pruritus on HS patients’ daily lives may be beneficial.

In conclusion, patients in this study with Hurley stage III HS reported high levels of daily pain and QoL impairment and many individuals reported self-directed use of over-the-counter treatments and CAMs to manage their pain. Most patients expressed a desire for increased counselling on pain management options. Therefore, physicians involved in the care of HS patients should consider implementing routine counselling on pain management into their clinical practices, especially for patients with severe HS. Strong interdisciplinary collaboration between primary care providers, dermatologists, and chronic pain specialists is essential to address the unmet need for pain management in the HS patient population.

## Supplemental Material

Table S1 - Supplemental material for Understanding Hurley Stage III Hidradenitis Suppurativa Patients’ Experiences With Pain: A Cross-Sectional Analysis Click here for additional data file. Supplemental material, Table S1, for Understanding Hurley Stage III Hidradenitis Suppurativa Patients’ Experiences With Pain: A Cross-Sectional Analysis by Leah Johnston, Elaine Dupuis, Lauren Lam and Susan Poelman in Journal of Cutaneous Medicine and Surgery
