# Supplementary material for: Understanding Hurley Stage III Hidradenitis Suppurativa Patients’ Experiences With Pain: A Cross-Sectional Analysis
Source: J Cutan Med Surg. 2023 Jul 25;27(5):487–92. doi: 10.1177/12034754231188452 (PMC10616983; doi:10.1177/12034754231188452)
Supplement: Table S1 - Supplemental material for Understanding Hurley Stage III Hidradenitis Suppurativa Patients’ Experiences With Pain: A Cross-Sectional Analysis [file sj-pdf-1-cms-10.1177_12034754231188452.pdf]

Supplemental Table 1. Patient Characteristics, Clinical Features of HS, and HS Symptoms

Demographic Category	Responses	Frequency (%) or Numerical Data
Sex (n=10)	Females	8/10 (80%)
	Males	2/10 (20%)
Age (n=10)	Mean	38.2 years
	Median	37 years
	Range	28-51 years
Reason for Clinic Visit (n=10)	New Patient Consultation	0/10
	Routine Follow-Up	6/10
	Fit-In for Flare-up Management	4/10
Age at Onset of HS (n=10)	Mean	19.6 years
	Median	17 years
	Range	8-44 years
Total Duration of HS (n=10)	Mean	18.7 years
	Median	21 years
	Range	2-34 years
Time from Onset of Symptoms to Diagnosis of HS (n=9)	Mean	11.2 +/- 6.7 years
	Median	10 years
	Range	1-22 years
Location(s) of HS (n=10)	Axilla	8/10
	Inframammary folds/chest	7/10
	Groin	9/10
	Buttocks	5/10
	Inner thighs	2/10
	Neck	1/10
	Abdominal pannus	3/10
Total Number of Affected Areas (n=10)	Mean	3.5
	Median	3
	Range	2-5
Pain (n=10)	Yes	10/10 (100%)
	No	0/10 (0%)
Pruritus (n=10)	Yes	10/10 (100%)
	No	0/10 (0%)
Frequency of HS Flare-Ups (n=10)	Daily	0/10 (0%)
	Weekly	5/10 (50%)
	Weekly to monthly	2/10 (20%)
	Monthly	2/10 (20%)
	Every few months	1/10 (10%)

HS, hidradenitis suppurativa.

Supplemental Table 2. HS Patients' Previous Medical Therapies, Treatments for Pain, and Previous Experiences Seeking Care from Physicians for Pain Management

Survey Question	Responses	Frequency (%)
Previous discussions with healthcare provider regarding pain management?	Yes	3/10 (30%)
	No	7/10 (70%)
Do you wish you had received more information on pain management from your healthcare provider?	Yes	9/10 (90%)
	No	1/10 (10%)
Previously prescribed pain medication by a healthcare provider?	Yes	3/10 (30%)
	No	7/10 (70%)
Prescribing Physician Specialty	Plastic Surgery	2/10 (20%)
	Rheumatology	1/10 (10%)
Type of Prescription Treatment for Pain	Opioids (short-term, post-surgical pain management)	2/10 (20%)
	Gabapentinoids	1/10 (10%)
Referrals for Pain Management	Medical Cannabis	1/10 (10%)
Routine use of over-the-counter pain medication?	Yes	8/10 (80%)
	No	2/10 (20%)
Routine use of complementary and alternative therapies?	Yes	7/10 (70%)
	No	3/10 (30%)
Specific Complementary and Alternative Therapies	Cannabis Products	6/10 (60%)
	Dietary Supplements	4/10 (40%)

HS, hidradenitis suppurativa.

Supplemental Table 3. Previous Medical, Laser and Surgical Treatments for HS, Previous Treatments for HS-Related Pain and Self-Ratings of Effect on Disease Activity and Pain

**Medical Treatment**	**Change in HS Disease Activity**		**Change in Pain (Mean +/- SD, range)**	
	**Mean +/- SD (Range)**	**Median**	**Mean +/- SD (Range)**	**Median**
Topical Antibiotics (n=10)	1.5 +/- 0.5 (1-2)	1.5	1.3 +/- 0.5 (1-2)	1
Topical Resorcinol (n=7)	1.9 +/- 0.9 (1-3)	2	1.6 +/- 1.0 (1-3)	1
Zinc Supplement (n=7)	1.4 +/- 0.7 (1-3)	1	1.3 +/- 0.7 (1-3)	1
Topical Antiseptic Washes (n=7)	1.9 +/- 1.0 (1-4)	2	1.9 +/- 1.0 (1-4)	2
Oral Antibiotics (n=9)	1.8 +/- 0.8 (1-3)	2	1.6 +/- 0.7 (1-3)	1
Intravenous Antibiotics (n=6)	2.3 +/- 1.4 (1-4)	2	2.2 +/- 1.2 (1-4)	2
Oral Anti-Androgens (n=6)	2.5 +/-1.1 (1-4)	3	2.5 +/-1.1 (1-4)	3
Intralesional Corticosteroid Injections (n=5)	2.8 +/- 1.0 (1-4)	3	2.8 +/- 1.0 (1-4)	3
Laser Hair Removal (n=2)	NC	NC	NC	NC
Botulinum Toxin Injections (n=1)	NC	NC	NC	NC
Biologics (n= 9)	2.6 +/- 1.2 (1-4)	2	2.6 +/- 1.3 (1-4)	3
Incision and Drainage (n=5)	3.6 +/- 0.8 (3-5)	3	4.0 +/- 0.6 (3-5)	4
Deroofing Surgery (n=5)	3.4 +/- 1.6 (1-5)	4	3.6 +/- 1.5 (1-5)	4
Wide Local Excision (n=1)	NC	NC	NC	NC

**Pain Treatment**	**Improvement in Pain**	
	**Mean +/- SD (Range)**	**Median**
Topical Lidocaine Creams (n=3)	2.0 +/- 1.0 (1-3)	2
Hot Compresses (n=7)	2.6 +/- 0.5 (2-3)	3
Cannabis Products (n=6)	2.3 +/- 0.7 (1-3)	2.5
Acetaminophen (n=8)	1.9 +/- 0.6 (1-3)	2
NSAIDs (n=8)	2.0 +/- 0.7 (1-3)	2
Oral Gabapentinoids (n=1)	NC	NC
Opioids (n=2)	NC	NC

HS, hidradenitis suppurativa; SD, standard deviation.
NC = Not calculated due to small sample size (≤ 2)

Supplemental Table 4. HS Patients' Scores on Standardized Pain and Quality of Life Rating Scales

**Pain Rating Scale**	**Mean +/- SD (Range)**	**Median**	**Severity Category/Impact on QoL**	**Frequency (n, %)**
Worst Pain in Last 24 Hours, NRS (n=10)	6.3 +/- 2.5 (2-9)	8	None (0)<br>Mild (1-3)<br>Moderate (4-6)<br>Severe (7-10)	0 (0%)<br>2 (20%)<br>2 (20%)<br>6 (60%)
Least Pain in Last 24 Hours, NRS (n=10)	3.8 +/- 2.4 (0-7)	4	None (0)<br>Mild (1-3)<br>Moderate (4-6)<br>Severe (7-10)	1 (10%)<br>4 (40%)<br>3 (30%)<br>2 (50%)
Average Daily Pain, NRS (n=10)	4.9 +/- 2.4 (0-8)	5	None (0)<br>Mild (1-3)<br>Moderate (4-6)<br>Severe (7-10)	1 (10%)<br>2 (20%)<br>5 (50%)<br>2 (20%)
Current Pain, NRS (n=10)	4.9 +/- 2.9 (0-9)	4.5	None (0)<br>Mild (1-3)<br>Moderate (4-6)<br>Severe (7-10)	1 (10%)<br>2 (20%)<br>4 (40%)<br>3 (30%)
DLQI (n=10)	19.5 +/- 8.2 (5-29)	19	None (0-1)<br>Small (2-5)<br>Moderate (6-10)<br>Large (11-20)<br>Extremely Large (21-30)	0 (0%)<br>1 (10%)<br>0 (0%)<br>4 (40%)<br>5 (50%)
BPI Interference Items (n=10)	5.6 +/- 3.0 (0.6-10)	5	Low (1-4)<br>High (5-10)	4 (40%)<br>6 (60%)
**SF-MPQ-2 Score Domains**			**Mean +/- SD (Range)**	**Median**
Total Score (n=10)			3.4 +/- 2.1 (0.6-6.3)	3.2
Continuous Domain (n=10)			3.1 +/- 2.1 (0.3-6.3)	3.0
Intermittent Domain (n=10)			3.5 +/- 2.2 (0.3-6.0)	4.6
Neuropathic Domain (n=10)			2.9 +/- 1.9 (0.3-6.0)	2.7
Affective Domain (n=10)			4.4 +/- 4.1 (0.0-10.0)	3.5

HS, hidradenitis suppurativa; NRS, Numerical Rating Scale; DLQI, Dermatology Life Quality Index; BPI, Brief Pain Inventory; SF-MPQ-2, Short-Form McGill Pain Questionnaire 2; QoL, Quality of Life.

Supplemental Table 5. HS Patients' Subcategory Scores on the SF-MPQ-2

Individual SF-MPQ-2 Pain Descriptors	Mean +/- SD (Range)	Median
Throbbing Pain (n=10)	4.5 +/- 3.0 (0-8)	4.5
Shooting Pain (n=10)	4.9 +/- 3.4 (0-10)	5.5
Stabbing Pain (n=10)	5.1 +/- 3.2 (0-10)	6.0
Sharp Pain (n=10)	4.8 +/- 3.5 (0-10)	5.5
Cramping Pain (n=10)	0.8 +/- 1.5 (0-4)	0.0
Gnawing Pain (n=10)	0.6 +/- 1.1 (0-3)	0.0
Hot-burning Pain (n=10)	3.4 +/- 3.4 (0-10)	2.0
Aching Pain (n=10)	3.4 +/- 4.0 (0-9)	1.0
Heavy Pain (n=10)	2.3 +/- 3.0 (0-8)	0.5
Tender (n=10)	7.2 +/- 3.1 (2-10)	8.5
Splitting Pain (n=10)	2.7 +/- 3.4 (0-9)	1.0
Tiring-exhausting (n=10)	5.1 +/- 4.7 (0-10)	6.0
Sickening (n=10)	3.9 +/- 4.1 (0-10)	2.0
Fearful (n=10)	4.6 +/- 4.7 (0-10)	4.5
Punishing-cruel (n=9)	3.6 +/- 5.5 (0-10)	0.0
Electric-shock Pain (n=10)	1.9 +/- 2.8 (0-7)	0.5
Cold-freezing pain (n=10)	0.2 +/- 0.6 (0-2)	0.0
Piercing (n=10)	1.8 +/- 2.7 (0-8)	0.0
Pain Caused by Light Touch (n=10)	5.7 +/- 3.3 (1-10)	5.5
Itching (n=10)	5.8 +/- 3.8 (1-10)	6.5
Tingling or 'pins and needles' (n=9)	1.8 +/- 2.5 (0-6)	1.0
Numbness (n=9)	0.9 +/- 1.3 (0-3)	0.0

SF-MPQ-2, Short-Form McGill Pain Questionnaire 2.
